# How do you play that makerspace game? An ethnographic exploration of the habitus of engineering makerspaces

**DOI:** 10.1007/s00163-022-00393-0

**Published:** 2022-08-12

**Authors:** Melissa W. Alemán, Megan E. Tomko, Julie S. Linsey, Robert L. Nagel

**Affiliations:** 1 School of Communication Studies, James Madison University, Harrisonburg, VA 22807 USA; 2 Pittsburgh, PA USA; 3 George W. Woodruff School of Mechanical Engineering, Georgia Institute of Technology, Atlanta, GA USA; 4 Department of Engineering, James Madison University, Harrisonburg, VA 22807 USA

**Keywords:** Ethnography, Makerspace, Habitus, Communication, Inclusivity

## Abstract

Drawing upon Bourdieu’s conceptualization of habitus, this ethnographic study explores the cultural bases guiding engineering makerspaces at a public university in the United States. Students carry forms of capital that impact their entry into these learning spaces, over time becoming disciplined in the “game” of makerspaces as they accumulate capital through everyday talk and storytelling. Communication constructs the makerspace habitus as students (1) move from outsider to insider as they acquire forms of capital; (2) negotiate a habitus characterized by tensions of access vs. exclusivity; (3) learn to use the vocabularies of innovation and creativity; and (4) cultivate supportive making communities. Findings point to the critical role of intentional communication and space design in cultivating inclusive makerspace cultures.

Calls to foster innovation correspond with the rising popularity of makerspaces on university campuses, particularly in the context of engineering education (Barrett et al. 2015; Rosenbaum and Hartmann 2017). Further, educators have argued that makerspaces have the potential to broaden participation in learning (Pines et al. 2015), a largely untested assumption. On the contrary, research indicates that makerspaces struggle to cultivate a culture of inclusivity, particularly for women (Roldan et al. 2018). For example, Tomko et al. (2021) qualitative study of women’s pathways into makerspace communities of practice found that girls and women confronted numerous barriers to participation, including forms of hostile and benevolent sexism and gendered assumptions about making. Further, Frank et al. (2020, 2021) research collaborating with the Diné of the Navajo Nation illuminate the cultural biases and limitations of normative White, Western models of making that govern many engineering programs. Studying the processes by which makerspace cultures are constructed in such normative environments is also critical to beginning to thoughtfully both *un-do* and *re-make* such spaces toward broadening participation.

We argue that to broaden participation and enhance the diversity of student populations benefiting from makerspaces, we must first understand the nuanced relations that construct and reproduce makerspace cultures. To do that, we draw upon Bourdieu’s theory of practice as a framework for examining the *habitus* of makerspaces. Bourdieu’s conceptualization of habitus provides a valuable framework for examining how communication in academic spaces both constructs and disrupts systems of inequality. Indeed, habitus has been used broadly as a framework to study educational systems at large (Edgerton and Roberts 2014), understand disciplines such as design (Gray 2013), architecture (Payne 2015), and engineering (Devine 2012; Foor et al. 2007; Mendoza et al. 2012), and critically interrogate the reproduction of culture in campus organizations (Workman 2001). Further, students’ experiences with makerspaces are shaped by dynamics of *awareness*, *knowledge*, and *access* (Whyte 2017)—all of which require what Bourdieu refers to as *capital* (Agbenyega and Klibthong 2015). Thus, Bourdieu’s framework offers a vocabulary for describing students’ experiences of an engineering makerspace and how that culture is reproduced through communication.

This ethnographic study reveals student experiences of the habitus of engineering makerspaces at a historically-White, regionally-focused, comprehensive mid-Atlantic university in the United States. Specifically, students’ reflections on norms for interactions and making in makerspaces reveal challenges and opportunities for inclusivity. The study highlights the role of communication in both enabling and constraining inclusive makerspace cultures and showcases the contributions that analyses of communication and culture offer in understanding such challenges.

## Makerspaces

Makerspaces, “a physical location that serves as a meeting space for a ‘maker community’ and houses the community’s design and manufacturing equipment” (Wilczynski 2015 p. 2), have risen exponentially worldwide (Lou and Peek 2016). For users, makerspaces are “more than just a room full of tools” (Tomko et al. 2018); interviews with students who frequent makerspaces show that students perceive them as communities supporting personal development through design exploration, community building, leadership, and individual expression (Tomko et al. 2019). Thus, makerspaces often considered a community of practice (Halverson and Sheridan 2014; Tomko 2019), cultivate opportunities for intentional and formal, as well as unplanned and informal interactions (Novak 2019). These different forms of interaction are often guided by programming and curriculum (e.g., Harron and Hughes 2018) and are situated within open, unstructured spaces (Toombs et al. 2014).

A preponderance of literature on makerspaces focuses on practical concerns, such as strategies for creating and building spaces, rather than the learning processes that happen within the space and the outcomes of makerspace use (e.g., Blacklock and Claussen 2016; Martinez and Stager 2013). Thus, the impacts of makerspaces on student learning are not well documented. In a recent review of the literature published through the *American Society of Engineering Education,* Weiner et al. (2018) found that just 5 out of 68 papers “made explicit and repeated references to Learning Sciences concepts, terminology, or theoretical frameworks” (pp. 9–10), indicating that significant work is still required to understand how academic makerspaces impact students’ education. Student engagement in makerspaces, though, has been demonstrated to increase design self-efficacy (Hilton et al. 2020) and is believed to also increase students’ creative confidence (Slåttsveen et al. 2017). Tomko (2019) further found that makerspace engagement develops a wide range of students’ intellectual, interpersonal, and intrapersonal proficiencies. Additionally, students engaged in makerspaces acquire a deeper understanding of their engineering course work with an increased motivation to learn as well as a shared sense of belonging as users of makerspaces (Nadelson et al. 2019).

While the specific impacts on student learning remain understudied, makerspaces are being developed in educational settings in a variety of campus venues (Barrett et al. 2015; Rosenbaum and Hartmann 2017) such as libraries (Mann 2018), STEM departments (Galaleldin et al. 2016), and dormitories (Roldan 2019). With the goal of increasing access to these spaces, makerspace administrators and managers are developing, applying, and testing different strategies ranging from online platforms to facilitate engagement (Slåttsveen et al. 2017) to student organization nights (Levy et al. 2016), to training, arts-based workshops, and tours (Noel et al. 2016). Exploring the characteristics of existing, well-established makerspaces at academic institutions in North America, Galaleldin et al. (2016) found that makerspaces tend to be designed with a target mission and were resourced, staffed, and academically integrated based on this mission. This finding, though, contrasts with the more traditional definition of a makerspace outlined by Wilczynski (2015) as being “community-organized with the community determining its structure, function, programming, and funding” (p. 2).

Despite the democratic assumptions underlying advocacy of the maker movement (Dougherty 2012; Hatch 2014), literatures across numerous disciplines consistently note challenges in cultivating an inclusive culture. Barton et al. (2017) specifically assert that,

there is little evidence that the maker movement has been broadly successful at involving a diverse audience, specially over a sustained period of time. The movement remains an adult, white, middle-community pursuit, led by those with the leisure time, technical knowledge, experience, and resources to make. (p. 5)

Ethnographic inquiry of makerspaces—from libraries to community hackerspaces—has afforded rich insights of the importance of studying the cultural contexts that shape membership and participation in makerspaces (e.g., Toombs et al. 2014; Whyte 2017). For example, Barton et al. (2017) ethnographic study of community makerspaces sheds light on the complex and often invisible barriers that prevent entry and demonstrates the importance of broadening the definition of making, developing community, infusing purpose, and supporting students through mentorship. Their research reveals the complex, and often invisible barriers that may prevent youth from “crossing the layered boundary from club lobby … into the makerspace” (p. 33).

To develop more inclusive makerspace communities, Roldan et al. (2018) identify strategies such as “scaffolding involvement” to provide students with conduits of entry, exemplifying who represents “valued members” of the makerspace, encouraging “perspective taking” to understand interactions from others’ point of view, ensuring the “approachability” of makerspace leaders, removing the barriers that might prevent someone from “asking for help,” and finally, creating “official statuses” that indicate one has gained specific knowledge and skills. Further, *how* we study makerspaces is critical toward changing the conversation and broadening participation (Halverson and Sheridan 2014). Research should inquire about learning processes within makerspaces as key to democratizing practices, inviting us to broaden our ideas about what counts as learning to “legitimate a broader range of identities, practices, and environments—a bold step toward equity in education” (Halverson and Sheridan 2014 p. 503). Studying the culture of learning in makerspaces is a critical component of such scholarship. Broadening participation and enhancing diversity requires that we understand the nuanced relations that construct and reproduce academic makerspace cultures; thus, we use Bourdieu’s theory of practice as a framework for examining the *habitus* of makerspaces.

## Habitus

Pierre Bourdieu’s social theory has been widely used as a framework in education research examining inequality (Edgerton and Roberts 2014; Edgerton et al. 2013). Educational systems, according to Bourdieu, are the primary sites for the reproduction of social inequality over time, and his concept of habitus provides a valuable heuristic framework for examining the cultural features that can serve to reproduce inequality in engineering makerspaces. “*Habitus* describes a construct that is both individual/psychological and social, and through this individual-to-group relationships, defines a given culture or set of social norms” (Gray 2013 p. 197, emphasis in original). While Bourdieu (2005) conceptualized habitus as dispositions shared with others and acquired through social conditioning, habitus is “embodied” through ongoing social interactions, ultimately reproducing patterns of differentiation, and thus, dominance between groups of people (Holton 2015). The concept of habitus allows educational researchers to look at the intersections between the social and the psychological to better understand how educational environments are reproduced and internalized.

Bourdieu’s theoretical framework draws upon the interrelated concepts of *capital, field,* and *doxa* to undrstand how social inequity is reproduced. There are several forms of capital within Bourdieu’s framework; of which, we explore three: *cultural, social,* and *symbolic capital*. According to Asimaki and Koustourakis (2014), “(c)ultural capital is composed of a body of symbolic goods and represents significant symbolic resources” (p. 124) that might include tangible goods and credentials (e.g., previous making experience). Social capital refers to one’s position within a complex web of group relationships (Asimaki and Koustourakis 2014), thus referencing the broader relationships that exist in a given culture, such as gender, that are often shaped by implicit biases. Symbolic capital describes access and use of cultural vocabularies and languages (Asimaki and Koustourakis 2014), such as technical vocabularies.

Bourdieu’s concept of *field* is also important to understanding the habitus of makerspaces, as it is ‘analogous to the “rules of the game”’ (Gopaul 2015 p. 76). By “rules of the game,” Bourdiesian scholars mean that participants learn how to navigate the complex social positions within that landscape, including structural relations of power within institutions (e.g., gender and race) and the acquisition of capital needed to succeed. Thus, fields are the contexts in which interactions among participants occur and may be physical locations (e.g., a fabrication lab) or discursive ones (e.g., engineering discipline). Like a game, players in the field work to uphold their position in the game accumulating various forms of capital. Capital becomes an important resource for reproducing the habitus of makerspaces, and thus “(a) student’s ability to understand the rules and play of the game is a result of accumulations of … *capital*” (Foor et al. 2007 p. 106).

To understand the development of capital necessary to successfully access and fully participate in academic makerspaces, we pose the following research question:

RQ1:What types of capital are perceived as important by students to participate in an engineering makerspace?

Capital might include permission to use particular makerspaces or the equipment therein, knowledge and skills to use the equipment, mentoring relationships with faculty or leadership positions, or even cultural knowledge from family members who are engineers. Fears, anxieties, and violations point to important kinds of capital needed to effectively ‘play’ in makerspaces, and thus, *capital becomes critical toward developing the campus-wide desired culture of innovation and creativity*.

Throughout this ethnographic examination, parallels are drawn between students’ tacit understandings of the “rules of play” and learning the “rules of play” associated with the culture of engineering programs, thus we look at the particular dispositional features of the culture of makerspaces (habitus), the various “rules of the game” (Gopaul 2015 p. 76) in which makerspace culture is played out (fields), and ultimately the common sense or taken-for-granted assumptions that guide behavior in those spaces (doxa).

RQ2:How do students experience a makerspace *habitus* (culture)?

RQ2a: What are the characteristics of the *fields* (“rules of play”) that students learn to participate in a makerspace habitus?

RQ2b: What are the *doxa* (taken-for-granted assumptions) that inform perceptions and behavior in a makerspace habitus?

Makerspace habitus is reproduced through the relationships between and among students, faculty, and staff participating in both informal and formal practices of making and is structured within the broader discourses of engineering education. In describing the features of makerspace habitus, we offer the challenges associated with accumulating capital and learning the “rules of play” that have the potential to explain inequities faced by women, first generation, and Black, indigeneous and people of color (BIPOC) students. Identifying the manner in which makerspace culture is reproduced via communication is critical to beginning to thoughtfully re-make such spaces in a manner that opens up, rather than closes down, their cultural accessibility to all students and allows institutions to realize their broader cultural goals.

## Field site: engineering and multidisciplinary makerspaces

This study occurred at a small, undergraduate-only engineering program (~ 450 majors) housed at a much larger (~ 21,000 students) comprehensive predominantly White university. Students coming into this program are typically attracted to the University by either the University culture, the open-enrollment process for engineering, or the flexibility afforded by the program’s single B.S. in Engineering (i.e., no disciplinary sub-divisions of engineering are offered).

Despite the program’s size, engineering was leading university-wide innovation efforts, carefully considering the design and remodeling of its makerspaces to include multiple flexible project labs, a 3d visualization studio, a fabrication studio, a machine shop, a high-bay, and design studios for each academic cohort. Flexible student work spaces and design studios are housed near faculty offices to increase the sense of community and provide a sense of “we’re in this together” concerning “learning engineering.” Flexible spaces, design studios, and the 3d visualization studio are equipped with easy-to-stack and store seating, tables on wheels, and wireless, short-throw projection systems. The 3d visualization studio at the time was equipped with six fused deposition printers (two high-end and four hacker-friendly) one poly-jet printer, and two stations for 3d scanning. The machine shop is equipped with tools for mostly metal work: manual lathes and vertical knee mills, box and pan breaks, bender, shear, four-axis CNC mill, horizontal and vertical bandsaws, sanders, and grinders. The fabrication lab is equipped for woodworking and most students’ first and second-year project work: metal top workstations, horizontal bandsaw, shopbot CNC router, drill presses, belt/disc sanders, and assorted hand tools including jigsaws, grinders, driller/drivers/ wrenches, files, hacksaws, taps and die sets. The high-bay provides a student-focused, semi-flexible, project-centered work space for multi-year projects that require outdoor access and/or vertical real-estate. At the time of this study, the high-bay housed two nascent competition teams being run as program-culminating engineering project experiences.

All engineering-housed spaces range in the degree of open access granted to students. Some spaces are considered open and readily accessible to all students (e.g., seminar rooms, flexible work spaces, and design studios), while for others (e.g., those with making and fabrication capabilities), access is granted only after students have taken and passed relevent safety trainings and quizes to ensure they understand the institutional rules for those spaces. Trainings increase in time commitments and oversight depending on the equipment housed in the spaces (e.g., the 3d visualization studio has online modules to complete, the fabrication studio requires completion of a two-hour long build with one-on-four oversight, and the machine shop requires completion of nearly eight hours of one-on-one training before acceptance into an apprenticeship program). While there are institutional rules that grant access and formal permission for students to enter and use these spaces, it is those informal and culturally constructed rules, as well as how students interpret and navigate the formal rules, that are of most interest to us for this project. While the makerspaces in the engineering program are not technically limited to use by engineering community members (students, faculty and staff), in practice due to formal and informal cultural rules, the spaces are only usable by members of the engineering community.

Researchers in this study participated in and observed both the multiple engineering makerspaces described above and the open cross-disciplinary makerspace. These makerspaces are highly integrated into their engineering curriculum, though it is important to note that students vary regarding the amount of time they spend in the makerspaces, often based on the roles they adopt in their team projects. Observed students were all engineering majors and were introduced to the makerspaces during their first year at the university through fabrication lab training. During fabrication lab training, students learn safety protocols associated with fabrication lab, and learn skills associated with basic wood fabrication including the use of the drill press, horizontal band saw, and hand tools. First-year students practice tool use through a common construction project, which at the time was building a trebuchet, and skills are reinforced through an Introduction to Engineering course-based project experience. At the time data were collected, second-year students participated in a year-long course in which they build a human-propelled vehicle for a client with a disability. This course has its own makerspace—“the bike lab,” and students use a variety of other makerspaces, such as the fabrication lab, during the course of the project. Formally, second-year students also completed an introduction to machining module, hands on training with a machinist and the use of both a vertical knee mill and a lathe. Following sophomore year, members of capstone teams, two-year project based learning which begins during students’ junior year, also use the makerspaces extensively. Once students have passed relevant trainings and safety tests as described earlier, they have permission granted to access locked spaces through their student ID cards (“swipe access”). In general, students do not formally receive training to use the digital fabrication equipment housed in the 3d visualization studio; instead, class projects, often coordinated with the makerspace staff, make use of these facilities. Additionally, many students arrive already familiar with the housed equipment (e.g., laser cutter, 3D printers, vinyl cutter) and/or gain exposure through engineering student organizations. These co-curricular engineering organizations are sometimes hosted in the makerspaces, as well as in community building making activities open to all students in the program. Students also use makerspaces, particularly ideational spaces, to do homework, study, and socialize.

We sought to understand the culture of makerspaces from a student’s perspective; therefore, following Institutional Review Board approval, undergraduate and graduate researchers trained in the processes of ethnographic methods conducted participant observation and ethnographic interviews (Lindlof and Taylor 2011) in and around the makerspaces on campus over a period of 18 months, engaging spaces at different times during the day and throughout the academic calendar. Student ethnographers took field notes to record their observations of what was happening in the spaces, ongoing interpretations of their interactions with others, and perhaps most insightful, captured reflections of their own experiences as engineering students in makerspaces. As is often practice in participant observation research, the student researchers conducted informal ethnographic interviews with people in makerspaces to solicit member reflections regarding their emerging interpretations, as well as to solicit a variety of perspectives from participants in makerspaces who occupy varied statuses and roles.

It is important to note that the student researchers themselves occupied different statuses with varying levels of familiarity and access to the makerspaces, yet all three were white cisgender women and men. The two undergraduate students, a white cisgender man and a white cisgender woman, collected ethnographic data during their junior and senior years in the engineering program. As it is a small program, the undergraduate researchers were relatively well known by students in their cohort and recognizable to other students in the program (perhaps not by name, however). The graduate student was a white cisgender woman near in age to the undergraduate students. She collected data while a visiting researcher at the university and developed a clear presence in the engineering department, though had more of an outsider status than the two undergraduate researchers. The student ethnographers were encouraged to participate and observe the spaces as they were going about their routine work as engineering students as well as during periods of high and low activity to interact with a wide range of student users. This enabled the student ethnographers to “test” out the rules of play in the makerspaces by pushing the boundaries of their own knowledge of those rules, learn rules as they would over the normal course of their educational experiences as engineering students, and affirm their understandings by interacting with other students and faculty.

## Analysis of ethnographic data

During the course of the ethnographic observations, the research team met weekly to share their field notes, discuss emerging interpretations, and participate in collaborative peer debriefings with the faculty team members and other student ethnographers. Weekly meetings allowed the team to identify points most salient in understanding makerspace culture as well as emerging research questions that would later guide observations and the content of the ethnographic interviews. In particular, themes related to access, accessibility, and inclusion emerged as important early features of makerspace culture that guided the foci of this project. Observations, reflections, and ethnographic interviews resulted in over 200 + pages of single-spaced typed field notes.

Based on the direction provided from emergent discussions among the research team, the field notes were further analyzed by the first author using QSR International’s N-Vivo qualitative data management software. Field notes were analyzed in multiple phases. The data were first to read multiple times in the immersion phase to provide a holistic sense of the ethnographic data, putting the data back together after the more emergent interpretations that unfolded during the data collection process (Tracy 2019). The first author next coded the data using processes of constant comparison (Glaser and Strauss 1967) during a primary cycle coding phase to describe ‘what was happening’ in the data (Creswell 2007; Tracy 2019), including in vivo codes in which the exact language of the participants was used to label data (Strauss 1987). Specifically, primary cycle coding focused characterizing makerspace experiences at the micro-level, coding for descriptors of physical features (e.g. locked doors, clutter), affective features of space (e.g. chaos, calm, unwelcoming), affective experiences of the user (e.g. anxious, excited, intimidated), types of interactions (e.g. helping, advising, questioning, storytelling, scolding), types of knowledge (e.g. tools, design, relational), and identities (e.g. gender, cohort), among others. As primary cycle coding serves to “open up” an understanding and interpretation of the data, these initial codes were numerous and demonstrated a complex experience of makerspaces not easily reduced to categorical labels. Yet this coding process enabled a close examination of the nuanced distinctions, particularly in the affective experiences expressed in the observations.

Third, first-level codes were organized using the heuristic framework of Bourdieu’s habitus, capital, field, and doxa to describe the larger features of makerspaces that emerged in the student ethnographers’ observations and reflections. This process was guided by the research questions, as we sought to better understand the perceived capital students need, as well as a broader description of the characteristics of the makerspace habitus. For example, we organized the student’s experiences navigating particular types of knowledge and resources, their identities, and their corresponding affective experiences through a lens of cultural, symbolic, and social capital. Further, because habitus is understood and reproduced through communication, the fields (“rules of the game”) and associated doxa (taken for granted assumptions) are organized around key codes for communication and normative relational expectations of the makerspaces.

The findings that follow are organized by the key themes that emerged over the course of the ethnographic study and presented in a holistic manner that reveal the students’ experiences of the engineering makerspace habitus. As such, exemplars from the ethnographers’ field notes and ethnographic interviews (qualitative data collected) serve as support for the identification of the capital, fields, and doxa of the makerspace habitus detailed below.

## Findings: habitus of an engineering makerspace

While technical knowledge and vocabularies (i.e., symbolic capital) are clearly important to student success in engineering and other academic enterprises, the social interaction that constructs, reproduces, and even resists the academic culture is important toward understanding how power relations are wielded, community is formed, and ultimately how learning happens. The makerspace habitus is characterized by the cultivation of a mindset of innovation and creativity shepherded through supportive interactional forms. Yet, the cultures and the contexts of the spaces themselves produce a tension of accessibility and exclusivity—cultivating privileged statuses and identities for some students who accumulate capital more readily than others or who enter the space with capital that others do not hold. The following describes the communicative processes through which the rules of the makerspace game are learned, practiced, and negotiated among students, faculty, and staff in the contexts of dominant discourses of the academic discipline of engineering at one university. Specifically, these ethnographic research findings are revealed below as a holistic story of the habitus of makerspaces at one university. Showcasing student experiences of habitus requires integrating findings regarding the forms of capital that students bring and acquire with the features of the fields and the doxa that inform student behavior.

The first research question sought to understand the varied types of capital that students bring to makerspaces. Perhaps most importantly, the ethnographic findings demonstrate that not all students enter the habitus of makerspaces with the same capital, meaning that some students feel greater comfort in makerspaces from the outset, while others have a steeper ramp to develop the capital needed to be successful. Several types of social capital shaped student’s experiences, including technological and relational. The opening section “Moving from Outsider to Insider” describes the processes and affective experiences in which students navigate makerspaces depending upon the kinds of capital they bring into the program and those collected along the way.

The second research question inquired about the student experience of a makerspace habitus (culture), including the features of the fields (“rules of play”) that students learn therein and the doxa (taken for granted assumptions) that inform both their perceptions and behaviors in a makerspace habitus. Ethnographic data demonstrate that students learn the fields through communication, and more specifically through the storytelling of others in the engineering makerspace habitus. In doing so, they come to learn how to negotiate tensions of inclusivity and exclusivity, incorporate codes of innovation and creativity as ways to communicate and learn the importance of supportive and collaborative communication within the makerspaces.

## Moving from outsider to insider—becoming disciplined in the makerspace habitus

Students bring forms of cultural capital to work in makerspaces. Some students arrive at the university from highly resourced primary and secondary schools and community environments where making was part of their learning experiences. Others do not share those experiences, and a university engineering program may be their first introduction to makerspaces. Further, gendered expectations in students’ families around tools and machinery may have equipped some students with a subjectivity that favored comfort and “naturalness” of the makerspace and innovation culture, and others not. Student ethnographers demonstrate that not everyone enters the making culture from the same vantage point. Ethnographers working on this project not only captured their experiences in and around makerspaces but also engaged with others illuminating that students both bring capital and develop capital as they learn the rules of makerspace cultures via interactions with others.

Anxieties, much like those revealed in the imposter syndrome that pervades academia, show up in initial encounters with makerspaces in some of the accounts of the women ethnographers in particular. Words such as ‘afraid,’ ‘fear,’ ‘anxious,’ and ‘intimidated,’ capture the affect of initiations with makerspaces:

Either way, I’m afraid to enter the makerspace. Afraid that they might figure me untrustworthy, if and when I tell other students about what I am doing. Afraid of just these spaces, for not knowing what to do in them.

This expressed fear is tied to the lack of technical knowledge as a source of symbolic capital. In another reflection, the same researcher further notes “I’m starting to feel intimidated by this room. I mean the guys [are] in the backroom and the fact that the room has a lot of equipment that I haven’t even seen or heard of.” In both cases, the student ethnographer expresses a fear of being unmasked as not knowing how to work and behave in a makerspace. Coupled with gendered experiences in childhood that distinguished her from her father, brothers, and construction workers, and we begin to see the differences in the social capital that students bring to makerspaces that may gender the making experience itself:

The music playing [in the makerspace] … reminds me of when we would have construction workers at my house growing up. Or even when my dad would be working on something around the house . . . For the construction workers, I usually stayed away from them. Didn’t want to bother them in their work. For my dad, it was pretty much the same, but if he needed my help then I would help. Mostly though, he’d ask my brothers*.*

The stories and experiences students bring to makerspaces inform their level of comfort, felt naturalness or foreignness of making cultures, and feelings of belonging. Thus, makerspaces may reproduce gendered landscapes, inviting students to draw upon other similar gendered spaces from their pasts, such as workshops.

In other instances, the students’ comfort is less about the *space*, but about the *people* working together in those spaces, leaving students feeling like outsiders in the spaces without another form of social capital—relationships:

I felt very uncomfortable in this situation because these are groups that have been working with each other all semester, and I felt like a complete outsider. I was not familiar with the dynamics of these groups or the projects.

In this case, the student does not understand how to enter the interactional scene of the team working in the makerspace, not knowing the rules of action and play, nor the roles that one would occupy in that space. This example showcases the interconnecteness between *capital* and *field* in understanding the habitus of makerspace, highlighting that makerspaces as not just physical places, but *relational* spaces as well. In other instances, the students describe feeling like an intrusion on already formed groups and the spaces that they “rightfully” occupied in the makerspaces, being mindful that remaining in those spaces alongside them may infringe upon their rights and territory. For example, one student expressed: “I know some teams had been assigned spaces, and I didn’t want to sit anywhere that would intrude on that space.” Indeed, the idea of “rights” and ownership of space was tied to the kinds of capital accrued prior to entering the space, impacting how they understood the fields of play in those spaces. This student’s reflection points to the social capital that is created over time as students become members of teams and establish themselves in relationship with known others in the spaces.

These uncertainties are not merely something that students bring to the scene, but they are produced through both verbal and nonverbal interactions among members, staff, and faculty. In one instance, a woman graduate ethnographer describes an interaction in which she felt the nonverbal gaze of a staff member questioning her presence in space:

I’ve gotten it before. I cannot quite describe it. It’s the look of someone believing that I don’t belong there. That I am using this machine and am not authorized to do so. It’s when someone who believes themselves to be of authority or superiority tries to inflict that authority and superiority on me. To reprimand me for doing something that I am not supposed to do. Because they don’t want people taking advantage of certain privileges and don’t want people to manipulate rules.

This same student, feeling “unknown” to an authority figure, reinforces the importance of “knowing people” and being known to others to feel both confidence and a sense of belonging in the maker environment. Upon recognizing another student in one of the new makerspaces in another instance, instead of apprehension, the same student felt excited about the possibility of working in that space. The people occupying the makerspaces shape how that space *feels*, cultivating the potential for learning:

Well, I felt at peace being there with someone that I knew. It really helped me to take out the fear that I usually feel when entering a new space. I felt safe and like this could be a place that I could work in.

Even seasoned makers reflected on the importance of community relations to feeling empowered and “safe” to work in makerspaces. Students described scanning the makerspaces for familiar faces and team members, even when their intent was to work independently.

Students became disciplined in the makerspace habitus through the tales and stories of other students. Such tales were ways to reinforce behaviors and forms of relation in the spaces, as Workman (2001) notes, “the rules of the habitus are illustrated through the stories told and accepted” (p. 430). One such oft-told tale is of the engineering students’ sophomore design project in which they design a human-powered vehicle for a client with unique needs in the community. Sophomores moving through the process look to older students to learn faculty expectations and to navigate the culture of the design studios. A White senior male engineering student noted an experience in which he was praising a group of sophomores for their good work while sharing his experiences with them:

[The sophomore students] asked me what year I was and then proceeded to ask me about my bike project. I repeated that our prototypes were not this in depth, but ‘good for them!’ and went on to tell them how I thought this project was the most rewarding thing I have ever been a part of. They asked why, and I told them my experience and how all the teams’ hard work had paid off when we got to show the bike to our client at the time.

This same student also showed that it was not only in sharing transformative tales but also being brutally honest with younger students that is important to them learning the cultural expectations:

With a half chuckle, I wished them luck [on their first-panel presentation]. The other upperclassman in the room overheard this and chimed in to wish them luck as well. They asked how bad it was going to be and a few others as well as myself explained our experiences . . . They seemed to listen very intently on each word coming out of my mouth like they were hoping for me to say ‘you’ll be fine. It is not a big deal,’ but spoke honestly, and I think in the end they appreciated that more.

Interactions between students that spun tales of faculty and coursework, panel presentations, design and capstone projects served to set up expectations that the major and the life within makerspaces were a series of rites of passage that one survived. Such tales not only serve to socialize, but also to reinforce one’s status in the community.

## Negotiating a habitus characterized by tensions of access vs. exclusivity

The makerspace is storied as an ideal learning environment—widely accessible, yet simultaneously exclusive. For example, in the following one student expresses this tension and the pull to the makerspaces:

But I start to feel that sense of exclusivity. It’s more like a yearning and desire to be in the space. You know that it is going to be for students, and we are just waiting for it. And then the door is open, but it is still a barrier. Like an invisible force that repels me from going in because I know that I am not allowed in there—at least that is what the sign says.

The tensions between access and exclusivity play out in broader campus conversations about the importance of innovation and creativity across disciplines and the role of makerspaces in students’ educational experiences. This has resulted in an infusion of funds into library makerspaces and other interdisciplinary makerspaces outside of engineering. At the same time, engineering students take pride in the status and identity associated with having access to the makerspaces in their department; the access affords a particular set of privileges that are important to accumulation of perceived status, a form of social capital, related to cultural and symbolic capital. Importantly, access and exclusivity are discussed in terms of training, safety, and resource allocation, and in many instances, students negotiate just what that access means to them—as one having access or not.

While locked doors rendered makerspaces inaccessible and exclusive, students resisted by propping open doors and opening doors for classmates, inviting them into the space. The perceived barriers were still powerful in keeping students out who were not formally granted access, even if the doors were open. For example, one student reflects “I don’t know if it is due to the fact that I was ‘locked out’ the first time or that the original time I came in I was with someone who knew a lot about the space, but this time I felt very nervous and uneasy about coming into the space.” After being locked out of a makerspace on a second visit, another student immediately felt the space was less welcoming, leaving her questioning whether the purposes were about the people or the equipment in the space:

I was surprised and immediately felt as though it was now an exclusive space. It lost some of its welcoming feeling it had last time I went. I tried to remember back to my first visit, and I recall that I was with one of the supervisors of the space and vaguely remember that he may have indeed swiped me in. Is this to keep people out or to protect the equipment inside?

The tools in the makerspaces were common to the students’ observations, noting favorite and unfamiliar machines, the “danger” and risks associated with some of the makerspaces, and the necessity of proper training on the equipment.

Students quickly learn that they must earn access to various makerspaces through training, an important form of symbolic capital. One ethnographer asked a student what he thought about the fact that key card access is required to get into the room that they were in, he wrote:

He replied that he thinks its [restricted access] is necessary because the room is “almost dangerous” since there is [sic] “no governing or rules.” Also, he mentioned that it helps filter the people allowed into those who really need it.

The student is highlighting multiple statuses here, as he points to a space that is unmonitored, thus being in the room affords a privileged status to use potentially dangerous equipment. He also is pointing out that not everyone “needs” to be in the makerspace, and that limited access not only allows people in but also *keeps others out*. This provides a critical socializing function as students come to take for granted who belongs in the space, and thus who can use the equipment, and who does not. Such taken-for-granted assumptions, doxa, arise from and result in particular ways of engaging the artifacts in the makerspace environment.

The tensions between access and exclusivity were not limited to the privileges afforded to enter a space, but also those resources allocated to those using the space. In one instance, for example, a student noted her surprise that another group in her cohort was granted a locker to store their project materials while hers was not. She writes, “I was surprised that they had a locker, since he is in the same class as me and we do not have one.” Thus, capital in makerspaces includes knowledge about and access to territorial and material resources that may be important to the work that students are able to accomplish in those spaces. In this case, she did not understand how that resource decision was made, leaving her to recognize the value of the resource, but without the accumulated knowledge to obtain it.

Finally, students, only men in this study, claimed their status with regard to spaces in the manner in which they entered those spaces, sometimes in a rather cavalier fashion. Whether it was skateboarding down the hall into a machine shop, or entering through a loading dock, students found ways to communicate to others their ownership and rights to those spaces, highlighted in the following example:

Suddenly, there was a banging on the loading dock garage door, which startled me as it echoed around the room. I went over to investigate and saw two of my classmates motion for me to open the door. I opened it just high enough for them to enter and greeted them as they came in. They thanked me and one mentioned that he always feels ‘really cool’ when he comes in that way.

How such rights and statuses are communicated may impact the experiences of others in that space, such as earlier examples suggest. This is particularly important to understanding the doxa about who is entitled to use and access makerspaces and who can break the rules of access. The doxa of access, the socialized assummptions about “who belongs in the makerspace,” was tied to one’s status as an engineer and presummed capability of handling “dangerous equipment,” and was often gendered masculine. This begs the question, to what extent might some students simultaneously want to belong to the exclusive engineering space, yet express shortcomings that leave them questioning their rights (even perhaps after they have been institutionally afforded)?

## Learning “innovation” and “creativity” as codes of communication

The concepts of innovation and creativity are hallmarks of engineering education (Forest et al. 2014), and are contemporaneously ubiquitous in conversations about what a twenty-first century education must look like for students in the United States to remain globally competitive. Yet, how do engineering students learn to use and reproduce this code in their everyday interactions in makerspaces?

Educate—Innovate—Prototype—Launch. It actually seemed pretty inviting. Those words that they used to describe [the space] weirdly put my mind slightly at ease. Those are the words that we as engineering students hear time and time again, so I suppose it was a sense of familiarity.

The code of innovation and creativity guides student’s expectations for what constitutes learning, the kinds of relations engendered in makerspaces, and how they identify as makers. Thus, the communication codes of innovation and creativity, and with it a set of vocabularies of making, are a form of symbolic capital that students accumulate.

*Organized chaos*. *Creativity is messy.* Students in this study drew upon central meanings of making and makerspaces in such terms. A senior engineering student reflected on the “security” he felt in one of the makerspaces:

Things are scattered around, chairs in the middle of the room, half-completed capstone projects fill the corners and any other vacant space has been filled with poorly erased white boards that show scratch work to thermal problems. Maybe it is because I am a senior and have spent a lot of time in this room or maybe it is because I have spent stressful nights as well as many, many laughs in this room but there is a sense of security in the chaos. If everything had a place and was always put away, I think I would be afraid to move anything in fear of ruining the well planned out organization that someone had clearly put time into.

Not all students described “messy” spaces as welcoming, though; others noted the difference between a space that was ‘dirty’ and looked unused, from those with projects scattered. Students tease out the meanings of inspiration in their accounts of the spaces, as “fast paced” and “exciting,” a combination of characteristics of the space and qualities of their interactions. One student noted how the makerspaces created a kind of energy that guided how students related, “The conversations in this place seem far less formal and much faster paced. The energy in the room feels much greater.” Makerspaces are locations in which students bring their designs “to life,’ as another student noted, “The space is exciting, as you get to have something you design ‘come to life’ in front of you.” Across these ways of talking, students talked about innovation as a kind of *life force.*

Students found their relationships with materials in makerspaces to be deeply meaningful—not only critical to understanding the design process but also themselves as engineers. A senior male engineer reflected on the excitement that was generated when coming to his capstone meeting with all the materials for their design project laid out on the table, enabling him to make his design concrete:

Someone had grabbed all of our materials from our locker and they were spread over the table. I found that having these materials present and holding them in my hands was enabling me to think about the physical design in a much clearer way, and got me very excited about the prospects.

Students conceptualized these codes in relation to the empowerment they felt in controlling the space and the materials themselves. Noting the modularity of the makerspace, one student interviewed appreciated the lack of administration of the space that enabled students’ freedom and control over how they work: ‘It is up to you how you would like to work. There is a lot of trusts since there are seldom people watching over the activities in the room.’

## Cultivating supportive making communities

Makerspace cultures were guided by supportive forms of communication and value for collaborating toward a larger goal, where cues may be taken from the design of the room itself. For example, the type and layout of the furniture in the room guides communication norms:

There are always people talking in here, and the tables here are not desks, so you face each other. No one is shushing you or telling you to be quiet.

The notion that students were part of a larger community helped form the identities of the students, and those identities were ranked and ordered by their status within the program (social capital). Status questions were common, marked by levels of access to makerspaces, types of projects students were designing, the coursework, and students’ experiential “firsts.” Students were organized and organized themselves in such cohorts, simultaneously signaling both a supportive community and differentiated statuses. Photos of students are organized by cohorts on departmental bulletin boards, and their presence is important for students as a signal of status.

While students recognized the differences in the status and ranking among the cohorts within the community, the communication forms of support featured heavily in their descriptions of how one engages in makerspaces. Supportive interactions, then, featured centrally in the mindset of making culture at this field site. Students characterized support in terms of variety meanings of helping: instructing, advising, and directing. Students supported other students in helping interactions, and faculty worked alongside students. In many cases, one student would be sharing knowledge that another needed:

I located the three people that were present in the side room where the virtual reality unit is located and greeted them. I recognized a friend of mine in engineering was among them and the instructor who ran the laser cutter during the last class. The instructor was in a conversation with the other individual who I did not recognize, so I squeezed into the crowded room and asked my friend what he was working on. He showed me a painting he had done earlier in the week and told me he was interested in using the laser to engrave it onto a surface but was struggling to get the program to do what he wanted it to. I told him that I had a program that would do what he wanted to do, so we exited the small room.

This example showcases the ways in which students bring their knowledge to bear to help another student accomplish a goal, cultivating a making community that is communal rather than individuated. Through participation, implicit rules such as *helping peers is an expected behavior*, are learned.

In ideational and tutoring spaces, supportive interactions were characterized by students moving around the room and assisting different people with projects and homework in an ad hoc manner. One mixed-use makerspace in particular was organized with multiple seating areas that included comfortable furniture and a work table frequented by students. A senior undergraduate student describes the ways in which students asked for help that ultimately cultivated community around mutual support:

… I was headed back to my seat when a male student on the couch with his computer on his lap and papers sitting on the armrests asked if I wanted to sit next to him (making it obvious that he would like my help on the same assignment I had just explained). I jokingly told him ‘no,’ then sat down and went through the process again.

Students described the makerspaces with meanings associated with “openness” making parallels to the collaboration that happens therein. One student noted, “There is an openness in the space that induces conversation as well as collaboration.” Such descriptions captured both the physical space and the configuration, but also the culture that was cultivated. Open relationships among students were considered crucial.

The researchers often observed supportive team interactions as seamless, like a dance, students often worked together in makerspaces through coordinated nonverbal interactions, such as the following:

The noise from the jigsaw went from loud in the hallway to deafening while inside. The conversations between the two working on the miniature house were brief and inaudible. When the saw was not running, all work in the space was happening very quietly with little [verbal] communication. It seemed they already had a plan to follow and they were simply executing it.

Such nonverbal coordination may be partially a product of the makerspaces themselves, particularly the machine and woodshops that are characterized by ambient noise and the whirring of machines and other tools, while ideational spaces where whiteboards are strewn around the room and alight with formulas and design ideas are characterized by a “buzz’” of talking. Here, making was often seen as a social activity, but also, a solitary one.

## Discussion

This ethnographic examination revealed the central role of communication in constructing the makerspace habitus, as well as the capital, field, and doxa that impact the experiences of students therein. The first research question inquired about the forms of capital that are needed for students to participate and thrive in makerspaces. Findings revealed that varied forms of social and symbolic capital were central in the students’ experiences of barriers, access, and belonging in the makerspaces. Symbolic capital featured heavily in students’ observations, particularly as students described a lack of capital related to knowledge about the equipment and their uses of spaces as a barrrier to participating. The perception that they lacked this knowledge was associated with anxiety, fear, and intimidation in the makerspaces, and limited their sense of belonging as rightful users in the makerspaces. Further, students coming to campus with significant differences in cultural capital (experiences in makerspaces and making environments in K-12, at home, and in the community) would also have greater symbolic capital (technical vocabularies and knowledge about making) then those that did not. As students acquired the technical vocabularies over the course of their study and their time as a student, their expressions of anxiety and fear regarding participating in the spaces became less prominent. This actual of symbolic capital worked in tandem with the social capital that students developed over time as they progress from first-year students on to seniors.

Social capital, such as gender expression and identity as well as status in the progam, was indeed important to student’s access and sense of entitlement to not just enter the makerspaces and use them, but also to claim ownership of those spaces. While men and women all expressed anxieties at some point in the study about their “right” to be in a makerspace, only women pointed to their status *as women* as a barrier to entry. This finding related to social capital may illustrate how gendered trends are reproduced in makerspace cultures themselves; a trend noted by Frank et al. (2020) as being perpetuated by the maker movement itself through its own MAKE: magazine. Gendered trends were noted as early as a decade ago, just as the university maker movement was beginning: 81% of makers self-report as men (Make/Intel 2012). Perhaps further limiting engagement, is the social construction of making and makerspaces as masculine (Meyer 2018), or perhaps even more so, the discipline of engineering itself (Hatmaker 2013; McIlwee and Robinson 1992). Even with these cultural barriers, through, women do engage; however, women choosing to engage in making likely face gender bias and unfair expectations about their abilities (Lam et al. (2019), as well as benevolent and hostile sexism (Tomko et al. 2021).

Gendered expectations of who belongs in a makerspace, the parameters of what constitutes making, and what tools are appropriately used by men and women are part of the doxa or cultural taken-for-granteds that are born of broader socializing messages about STEM (Archer et al. 2020). Such doxa guide the implicit assumptions made about who belongs in a makerspace, creating interactions where one’s right to be in a space may be questioned or contested by others. Combatting such assumptions regarding gender and making require intentional intervention in the curriculum and in the messages of faculty and staff to challenge limiting gendered norms and broaden participation.

It is important to note as a central limitation of this study that the makerspaces studied herein are based on a singular narrative of making; one that is also predominately white and western. This perspective has been demonstrated to be limited in broader applicability (e.g., developing social relationships, life, and culture as found when making is explored through an Indigenous lens), yet this is the definition most commonly adopted throughout the United States (Frank et al. 2020). Further, all of the student ethnographers were White cisgender men and women. These contextualizations limit our understanding of the nuanced ways in which gender, race, and cultural expectations of making intersect and result in forms of capital for some privileged members of the community but not others. With this limitation in mind, we believe that the processes revealed in the study illuminate the very codes that can serve to limit and exclude, as well as those that can open up opportunities for participation.

The distribution of other forms of capital in the field is tied to students passing required safety tests to gain access to otherwise locked makerspaces, as well as matriculation through the program that affords particular social statuses to juniors and seniors. The distribution of resources in the field is not always clear to the students, creating barriers to the accumulation of capital needed to thrive. In one instance, a woman student noted that she did not understand the mechanisms through with resources (in this case, a locker) were allocated to students for their use. She recognizes that such resources could save valuable time and energy for students working in the makerspaces for their senior projects. In this way, capital is directly connected to the field of the makerspace habitus, as students must learn *how* resources, knowledge, and certain statuses can be acquired to fully participate in the spaces.

The second set of research questions more broadly sought to examine the features of the habitus of makerspaces to showcase the ways in which field and doxa inform how students learn how to become “players” in the makerspaces. The findings reveal that students learn how to “play the makerspace game” as they (1) move from an outsider to an insider and become disciplined in the makerspace habitus as they acquire capital; (2) negotiate a habitus characterized by tensions of access vs. exclusivity; (3) learn to use the vocabularies of innovation and creativity as codes of communication; and ultimately, (4) actively cultivate supportive making communities. It is important as makerspace designers that we consciously keep in mind the fields through which makerspace users move from an outsider to an insider and become disciplined in the habitus of the makerspace so that we can identify practical interventions that can enhance students’ ability to understand how to participate in the community and how we as educators wish to intentionally shape makerspace habitus. A conscious decision is made when deciding whether to design for either access or exclusivity; fostering a culture of innovation and creativity requires design; and making a making community of practice requires cultivated support.

## Implications for practice

This work represents a snapshot of a singular moment in time in one engineering program undergoing change as a part of campus-wide innovation and entrepreneurship activities. The data were collected prior to the COVID-19 pandemic which closed university campuses, forced rethinking of community and altered the way we engage with academic makerspaces. Moving forward, as we reopen our communities and makerspaces, we call for the identification of educational practices related to making and makerspaces that can intervene to combat implicit biases, level access to capital, and reduce other barriers that limit access for women and BIPOC students. Further, we call on faculty and staff to understand students’ anxieties and fears as well as the communicative contexts that give rise to them; knowledge and understanding can provide educators with important information necessary for rethinking the design of makerspaces themselves as we re-engage our makerspaces such that we can serve to remedy inequities. The findings of the study offer a number of implications for makerspaces designers, coordinators, and educators to use as a starting point as we rethink and redesign.

### Cultivate student capital

Lab managers, faculty, and staff need to intentionally shore up the capital students need to participate in makerspaces and recognize other sources of capital that may not be recognized by dominant narratives of making. In doing so, they must also attend to the social capital (and lack thereof) that motivates and disables participation. First, educators must be aware of the different levels and types of capital that students bring to the makerspaces and that very well may be preventing their entry into the spaces, or feelings of belonging while there. Creating well-publicized workshops, tours, and tool trainings can serve to invite students into makerspaces with an explicit purpose, building comfort in the space and creating new knowledge about the facilities and tools. Further, students may not see their previous experiences, such as crafting and other DIY activities, as forms of capital to be used in makerspaces. Makerspaces can serve to highlight and legitimate these experiences through celebrating diverse forms of making and showcasing projects, such as fiber arts and paper crafts.

Second, educators need to intentionally build social capital that attends to (1) broader doxa that gender makerspaces masculine; and (2) the need for supportive social relationships that build a sense of belonging in makerspaces. Our findings specifically support Roldan et al. (2018) qualitative study of women engineers in makerspaces in a number of ways, as they found that women’s representation as mentors was important to compensate for the otherwise masculine environment, which ultimately builds students’ social capital. Further, social capital in the form of supportive relationships can be built by encouraging students to bring their friends who have not participated in the makerspace, creating points of entry for those who may not otherwise cross the threshold of the makerspace doorway.

Third, as students’ anxieties were largely associated with their perceived lack of symbolic capital, specifically knowledge of the equipments uses and functions, educators can scaffold information and training to serve the different needs of the students in the spaces. For example, providing clear signage regarding the uses and functions of equipment and areas of makerspaces, as well as having clearly identifiable lab technicians who are trained and approachable is key. Building making activities into first and second-year coursework can introduce students to the makerspaces and engaging in training and learning activities together with others, serving to remedy inequities in symbolic capital that may characterize any given cohort of students.

### Debunk myths about makerspaces

Makerspaces developers and managers need to interrupt and disrupt taken-for-granted assumptions (*doxa*) that create barriers to participation in makerspaces. Our findings show that even those who have been granted access to makerspaces have some anxiety and stress over perceptions of their legitimate right to work in the makerspaces, largely based upon assumptions grounded in cultural ideals about makerspaces. Thus, makerspace designers and managers should consider comprehensive strategies to intentionally disrupt such cultural assumptions. First, educators need to debunk the myth “think maker, think man,” by creating intentional messaging in the environment itself and through events. Research has shown that representation matters, thus hiring women and people of color as staff in makerspaces is important to disrupting conceptions of who belongs in a makerspace (Roldan et al. 2018). Second, in K-12 and higher education settings, educators can create community engagement projects serve to both builds out the cultural capital (access to making experiences) and build diverse communities of makers (see Barton et al. 2017). Third, to broaden understanding of what constitutes making, Educators in both college and K-12 contexts should develop making projects that appeal to a diverse group of students as entry points, such as e-textiles (Herron and Hughes 2019). Fourth, universities can build programming that connects making and mentorship.

### Leverage ideals of making that generate excitement

Makerspace developers need to leverage and make visible those taken-for-granted assummptions (doxa) about makerspaces that enable and excite student participation. Our findings showcase that students view makerspaces as a place in which creativity is enabled through “organized chaos” and in the excitement that students feel when a design “comes to life.” Educators can leverage this excited both in the creation of spaces that enable design thinking to meet making (e.g. Standford’s d-School and Northwestern’s Segal Design Institute) and curriculum that encourages students to work with complex and messy real-world problems. Further, projects that are client driven and involve community and corporate stakeholders can serve to build student engagement and generate intentional adoption of codes of innovation and creativity from our findings. Such community engaged and “messy real-world” projects enable students to leverage spontaneity and creativity, while simulatneously building new forms of capital, challenging narrow doxa about making, and empower students to see themselves as makers within a loosely structured environment that supports their varied identities.

### Promote storytelling

Narratives are one of the primary mechanisms through which norms and expectations are conveyed. Narratives, however, can construct norms of an exclusive, rather than an inclusive environment and reify relations of power (such as makerspaces as masculine). Thus, understanding the ways in which faculty and students participate in cultural storytelling in and about learning spaces, such as makerspaces, lends insight into the ways in which disciplinary cultures are unquestioned and reproduced in everyday practices. At the same time, educators and practioners can intentionally use storytelling as a means to cultivate more inclusive communities. We urge educators to promote student storytelling that assists newer students in understanding how to participate in makerspace *habitus*, how to acquire different forms of capital, and what to expect. Makerspaces, or departments that house them, can hold annual showcases that assist students in important sensemaking and storytelling about their making experiences that enable novice members to learn about the possibilities of making. Further, educators can facilitate student storytelling across cohorts in making-centered programs. For example, creating opportunities for junior and senior undergraduate students to tell their making journey stories to first-year and second-year students. Such storytelling would enable students early in their college career to hear about the joys and triumphs of the making experiences, learn different strategies for confronting challenges and hardship, and broaden students understanding of the scope of making. Stories such as these can serve to intentionally socialize newer members of making, and ultimately create the opportunity for more inclusive narratives of making.

The study of the culture of makerspaces is one of many important mechanisms toward efforts to increase the representation of women, first generation, and BIPOC students in engineering programs as it enables educators to (1) critically examine taken-for-granted assumptions that reveal biases of dominant ideas about making, (2) identify the forms of capital for both participating and thriving in makerspaces, and (3) realize and attend to the emotional experiences for students when they feel as if they do not belong in a makerspace culture. For example, understanding how students’ anxiety is experienced in connection to their capital can aid educators in developing strategies that elevate the value of students’ varied backgrounds and experiences in making and clearly communicating opportunities to develop new skills. Thus, the findings serve to demonstrate the value of this framework for understanding how barriers operate and the intentionality required of educators to cultivate makerspace cultures that do not narrowly reproduce the dominant narrative of making and of makerspace. Future research methodologies should adopt an intersectional approach to studying the makerspace habitus to better understand the nuanced ways that students experience and navigate makerspaces and to identify sources of capital and conceptualizations of making not yet revealed. Doing so would render race, ethnicity, gender, sexuality and class visible as social locations that shape and are shaped within makerspace cultures. Further, applied research can begin to develop and test the suggested interventions for reducing students’ anxieties and enhancing their sense of belonging in engineering makerspaces, particularly for women, BIPOC, and first-generation engineering students. Drawing upon those features of makerspace habitus that bolster success, such as supportive and open communities, while redressing the forms of capital that cultivate a feeling of exclusivity appear to be one important key to that effort.
